# Supplementary material for: Comparison of Glucose Metabolizing Properties of Enterobacterial Probiotic Strains In Vitro
Source: Nutrients. 2024 Aug 13;16(16):2677. doi: 10.3390/nu16162677 (PMC11357327; doi:10.3390/nu16162677)
Supplement: Supplementary file 1 [file nutrients-16-02677-s001.zip › nutrients-3129878-supplementary.pdf]

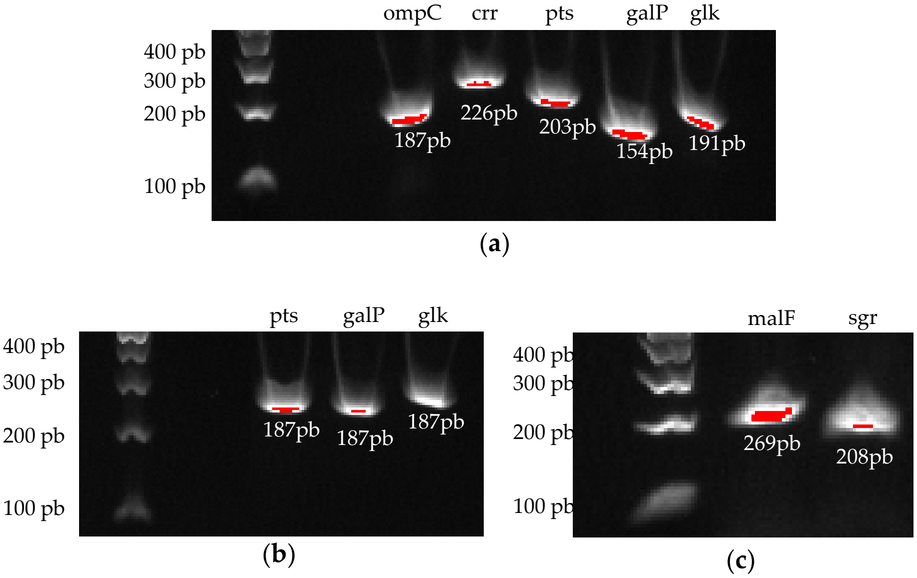

Figure S1. Detection of the PCR products of agarose gel. *E. coli* K12 (**a**) and *H. alvei* (**b**) and (**c**).

Table S1. PCR primers used in the study

Gene name	Sequence	Size	
E.coli K12			
OmpC	Fw: CGGCCTGAACTTTGCTGTTC	187 pb	
	Rv: ACGTTTGGAGCTGGGAGATCG		
crr	Fw: TTGGTCTGGCCGAAACATCA	226 pb	
	Rv: GTTTTCAGCAACCACCGCAT		
pts	Fw: TCACCACCATGGGCGTTATC	203 pb	
	Rv: AATGCCGAGGAATGCCAGAA		
galP	Fw: ATGCTCAACACGCTGGGTAA	154 pb	
	Rv: CGCGCAGTTTACGACCTTTC		
OmpC	Fw: ATTAGTCGGTGATGTGGGCG	191 pb	
	Rv: CGGTAATTGGGCAAGCGATG		
H. alvei			
malF	Fw: AGACCAATGACTGGGAAGCG	269 pb	
	Rv: TTCTAAACCTGCACCCGGAC		
sgr	Fw:AGCTCAGTACGCCAAATGCT	208 pb	
	Rv: TCTGTCGCGCTAAATGGGTT		
pts	Fw: ATCAGCCGTTGGTGAGTGAA	220 pb	
	Rv: GGGAAACCGCTTTGCAACAT		
galP	Fw: TGGTGTTATCGCTGGTGCTT	198 pb	
	Rv:AACAGGGACCCAACACAAA		
Glk	Fw: TGGCGGTTCCTATGCTCAAG	221 pb	
	Rv: TGGACGCAACACTTCAAGGA		
